# Intra-Peritoneal Administration of Mitochondrial DNA Provokes Acute Lung Injury and Systemic Inflammation via Toll-Like Receptor 9

**DOI:** 10.3390/ijms17091425

**Published:** 2016-08-30

**Authors:** Lemeng Zhang, Songyun Deng, Shuangping Zhao, Yuhang Ai, Lina Zhang, Pinhua Pan, Xiaoli Su, Hongyi Tan, Dongdong Wu

**Affiliations:** 1 Department of Intensive Care Unit, Xiangya Hospital, Central South University, Changsha 410008, China; zlmddzyx@gmail.com (Lm.Z.); dengsy2014@foxmail.com (S.D.); ayhicu1978@126.com (Y.A.); zln7095@163.com (L.Z.); 2 Department of Respiratory Medicines, Xiangya Hospital, Central South University, Changsha 410008, China; pinhuapan668@126.com (P.P.); Suli8779@163.com (X.S.); leopard2a1980@hotmail.com (H.T.); wdd6688@126.com (D.W.)

**Keywords:** lipopolysaccharide, mitochondrial DNA, acute lung injury, systemic inflammation, systemic inflammation

## Abstract

The pathogenesis of sepsis is complex. Mitochondrial dysfunction, which is responsible for energy metabolism, intrinsic apoptotic pathway, oxidative stress, and systemic inflammatory responses, is closely related with severe sepsis induced death. Mitochondria DNA (mtDNA) contain un-methylated cytosine phosphate guanine (CpG) motifs, which exhibit immune stimulatory capacities. The aim of this study was to investigate the role and mechanism of mtDNA release on lipopolysaccharide (LPS) induced acute lung injury (ALI) and systemic inflammation. Following LPS injection, plasma mtDNA copies peak at 8 h. Compared with wild-type (WT) mice, mtDNA in toll like receptor 4 knockout (TLR4 KO) mice were significantly decreased. MtDNA intra-peritoneal administration causes apparent ALI as demonstrated by increased lung injury score, bronchoalveolar lavage fluid (BALF) total protein and wet/dry (W/D) ratio; mtDNA injection also directly provokes systemic inflammation, as demonstrated by increased IL-1β, IL-6, high-mobility group protein B1 (HMGB1) level; while nuclear DNA (nDNA) could not induce apparent ALI and systemic inflammation. However, compared with WT mice, TLR4 KO could not protect from mtDNA induced ALI and systemic inflammation. Specific TLR9 inhibitor, ODN 2088 pretreatment can significantly attenuate mtDNA induced ALI and systemic inflammation, as demonstrated by improved lung injury score, decreased lung wet/dry ratio, BALF total protein concentration, and decreased systemic level of IL-1β, IL-6 and HMGB1. MtDNA administration activates the expression of p-P38 mitogen-activated protein kinases (MAPK) in lung tissue and specific TLR9 inhibitor pretreatment can attenuate this activation. Thus, LPS-induced mtDNA release occurs in a TLR4-dependent manner, and mtDNA causes acute lung injury and systemic inflammation in a TLR9-dependent and TLR4-independent manner.

## 1. Introduction

Severe sepsis, associated with systemic inflammatory response syndrome (SIRS) and multi-organ failure (MOF), remains a leading cause of death in intensive care units (ICUs) [1,2]. In China, the mortality rate from severe sepsis is 40.7% during the first 28 days and leads to health care costs of approximately 90,000 Yuan for each patient [3]. The pathogenesis of sepsis is complex, but a substantial number of studies indicate that mitochondrial dysfunction, which is responsible for energy metabolism, the intrinsic apoptotic pathway, oxidative stress, and systemic inflammatory responses, is closely related with severe sepsis-induced MOF and death [4,5,6].

In addition to disruption of energy production and initiation of the intrinsic apoptotic pathway, emerging investigations have also implicated the mitochondria as playing a critical role in the regulation of inflammation. During stress and injury, certain molecules released from impaired mitochondria function as danger-associated molecular patterns (DAMPs) [7]. The list of mitochondria-derived DAMPs includes reactive oxygen species (ROS) [8], mtDNA [9], *N*-formyl peptides [10], adenosine triphosphate (ATP) [11], cytochrome C [12], and carbamoyl phosphate synthetase I [13]. Recently, the role of mtDNA as DAMPs in inflammation initiation has garnered much attention.

Human mtDNA is a double-stranded circular molecule of 16.6-kb and contains 37 genes that code for two ribosomal RNAs, 22 transfer RNAs, and 13 polypeptides. MtDNA, similar to that of its bacterial ancestors, consists of a circular loop and contains a significant amount of un-methylated DNA present as CpG islands [14]. Under physiological conditions, the mtDNA encodes the mitochondrial respiratory chain complex associated protein. However, under a variety of critical conditions, especially after cellular damage and stress, mtDNA is generally released and plays an important role in the development of different kinds of inflammatory diseases, including rheumatoid arthritis [15], HIV infection [16], acute ischemic stroke [17], traumatic brain injury [18], acute liver failure [19], severe sepsis [20,21], and sterile SIRS [9].

A marked elevation in plasma mtDNA levels has been observed in animal hemorrhagic shock models [22,23]. In addition, studies of both sepsis patients and septic animal models have indicated that circulating free mtDNA fragments are increased in response to pathogens [24,25]. The plasma level of mtDNA is related to the markers of inflammation, severity of shock, and organ damage and mortality in septic shock patients [26]. Increased plasma mtDNA levels could be used as a predictor of outcome in severe sepsis patients [27]. Mitochondrial fragments, containing mtDNA, injected in the tail vein of mice can directly induce a systemic inflammatory response, acute lung injury, and neutrophil infiltration in the liver and kidney [9]. MtDNA is important for caspase-1 activation in response to LPS and ATP [24]. Similarly, mtDNA leads to activation of NLRP3 inflammasomes and mediates the secretion of IL-1β and IL-18 [14]. It was recently reported that mtDNA can enhance polymorphonuclear (PMN) adherence to endothelial cells, activate PMN-endothelial cell interactions and subsequently increase systemic endothelial permeability, leading to acute lung injury [28]. Shock-injured tissue might actively release mitochondrial debris, including mtDNA, which activates neutrophils through TLR9/p38 MAPK, leading to MMP-8/MMP-9 release and IL-6 and TNF-α hepatic accumulation [29]. Intra-tracheal administration of mtDNA in mice induced infiltration of macrophages, production of proinflammatory cytokines (including IL-1β, IL-6, and TNF-α), and activation of p38 MAPK [30].

Although it has been reported that mtDNA can activate both the TLR9/p38 MAPK and NLRP3/caspase-1 signaling pathways, the potential ability of intra-peritoneal administration of mtDNA to induce systemic inflammatory responses and acute lung injury is still not fully understood. In addition, the mechanism and kinetics of LPS-induced mtDNA release needs further study. In the current study, we hypothesize that local administration of mtDNA may lead to acute lung injury and systemic inflammation through TLR9, demonstrating the essential role of LPS-induced mtDNA release. In order to investigate this hypothesis, the effects of local administration of mtDNA on remote organ damage and systemic inflammation were examined in a murine endotoxemia model. Exploring the function of LPS-induced mtDNA release may further the current understanding of the mechanism of systemic inflammation and provide a novel therapeutic target for endotoxemia.

## 2. Results

### 2.1. LPS-Induced Circulating Mitochondria DNA (mtDNA) is Released in a TLR4 Dependent Manner

**Figure 1:**
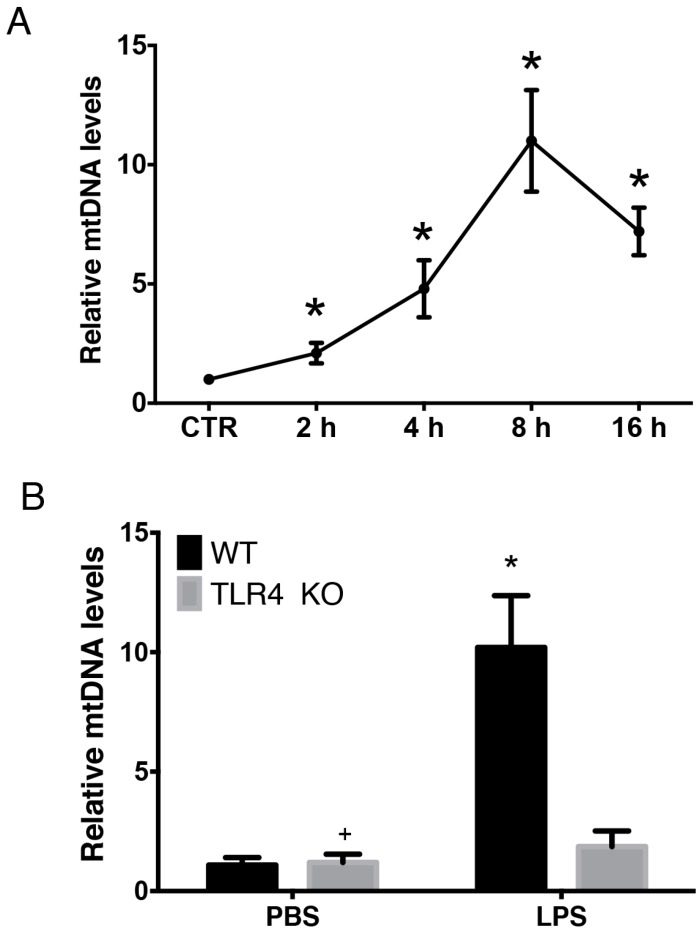
LPS-induced circulating Mitochondria DNA (mtDNA) release occurs in a TLR4 dependent manner. (**A**) Wild-type (WT) mice were given LPS (20 mg/kg) via intra-peritoneal injection. Plasma was collected 2, 8, and 16 h later for mtDNA copy quantification by Q-PCR. * *p* < 0.05 versus control group; (**B**) WT and matched TLR4 knockout (KO) mice were randomly assigned to groups and given either PBS or LPS via intra-peritoneal injection and plasma was collected for mtDNA quantification by Q-PCR. * *p* < 0.05 versus TLR4 KO/LPS group; + *p* > 0.05 versus TLR4 KO/LPS group. Eight mice were used in each set and data are mean ± standard error of mean (SEM) of three separate experiments.

LPS has been shown to induce mtDNA release [31]. LPS was administered to C57BL/6 mice via intra-peritoneal injection and plasma was collected at indicated time points for mtDNA quantification by Q-PCR in order to further explore the time course and mechanism of LPS-induced mtDNA release. As shown, LPS led to systemic mtDNA release in a time-dependent manner. There was a marked elevation in circulatory mtDNA as early as 2 h post-LPS administration and mtDNA copies peaked 8 h following administration of LPS and then gradually decreased (Figure 1A). To clarify the mechanism(s) involved in LPS-induced mtDNA release, both wild-type (WT) mice and matched TLR4 knockout (KO) mice were used. As demonstrated previously, TLR4 activation in sepsis leads to mitochondrial structure damage [31]. Thus, the role of TLR4 in LPS-induced mtDNA release was explored. Following LPS treatment, plasma mtDNA copies in TLR4 KO mice were significantly lower than in WT mice (*p* < 0.05) (Figure 1B). There was no increase in circulating mtDNA release in the TLR4 KO group following LPS administration (*p* > 0.05) (Figure 1B). Thus, LPS-induced circulating mtDNA release occurred in both a time- and TLR4-dependent manner.

### 2.2. Intra-Peritoneal Administration of mtDNA Leads to Acute Lung Injury (ALI)

**Figure 2:**
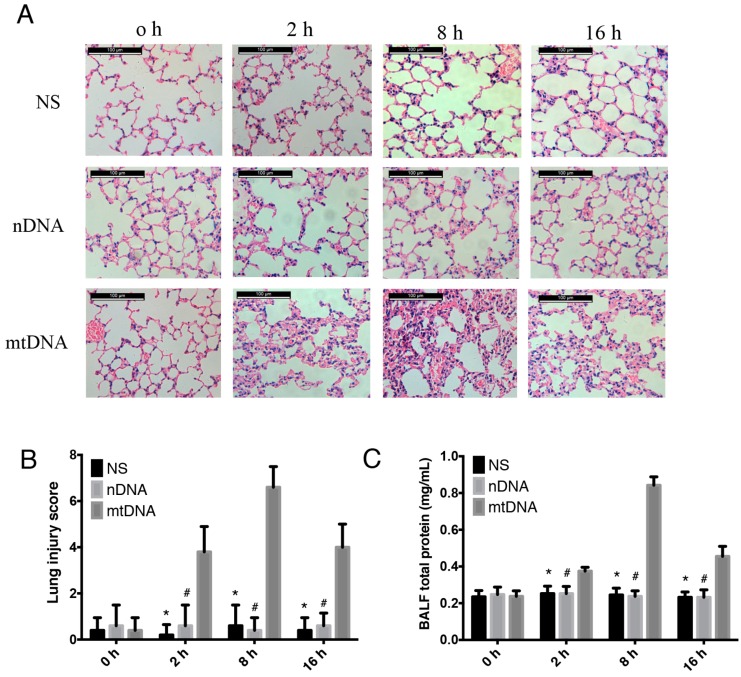

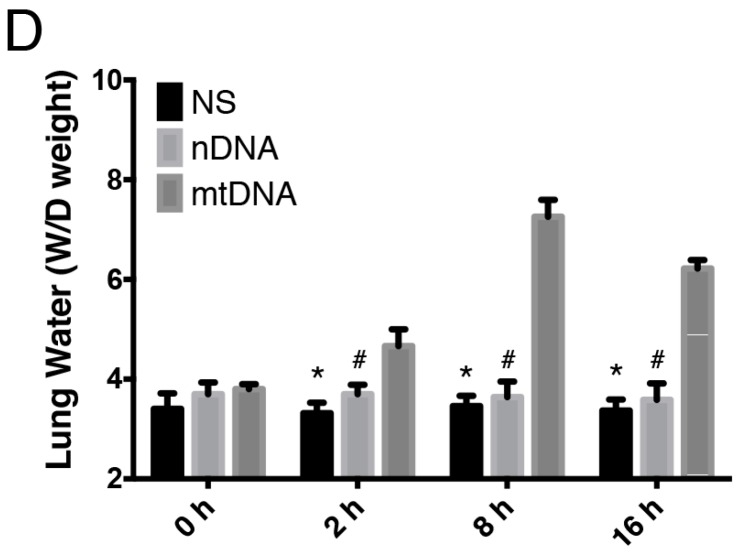
Intra-peritoneal administration of mtDNA leads to acute lung injury. WT mice were treated with NS, nuclear DNA (nDNA), or mtDNA via intra-peritoneal injection for 2, 8, and 24 h (*n* = 16/group, 8 for bronchoalveolar lavage fluid (BALF)). Acute lung injury was measure by hematoxylin and eosin (HE) staining (scale bar is 100 μm) (**A**) for lung injury score (**B**); BALF total protein concentration (**C**); and lung W/D ratio (**D**) were also analyzed. * *p* < 0.05 versus mtDNA group; # *p* < 0.05 versus mtDNA group. Eight mice were used in each set and data are mean ± SEM of three separate experiments.

mtDNA can increase endothelial cell permeability, which is essential in the pathogenesis of acute lung injury [28]. WT mice were treated with normal saline (NS), nuclear DNA (nDNA), or mtDNA via intra-peritoneal injection. Samples were collected at different time points (2, 8, and 16 h) in order to explore whether intra-peritoneal administration of mtDNA leads to remote end organ injury. As shown, the intra-peritoneal mtDNA administration group developed marked lung inflammation, hemorrhage, interstitial edema, and inflammatory cells were observed in most of the alveolar spaces (Figure 2A). The mtDNA group had a significantly higher lung injury histological score compared to the control and nDNA groups (*p* < 0.05) (Figure 2B). MtDNA intra-peritoneal injection causes typical symptoms of inflammation as early as 2 h, including ruffled fur, hunched posture, reduced activity to external stimuli, and shivering. These symptoms have been observed in 84.5% of the mice following 2–6 h post mtDNA administration (data not shown). Mice in the control group and nDNA groups did not exhibit signs of inflammation. The pulmonary tissue histological score (Figure 2B), the BALF protein concentration (Figure 2C) and lung wet/dry (W/D) ratio (Figure 2D) in the mtDNA group were also markedly increased as early as 2 h after mtDNA injection, peaked at 8 h and then gradually decreased; however, the values after the decrease remained significantly higher compared with the values for the nDNA and control groups. Thus, intra-peritoneal administration of mtDNA led to remote organ injury and acute lung injury.

### 2.3. Intra-Peritoneal Administration of mtDNA Leads to Systemic Inflammation

**Figure 3:**
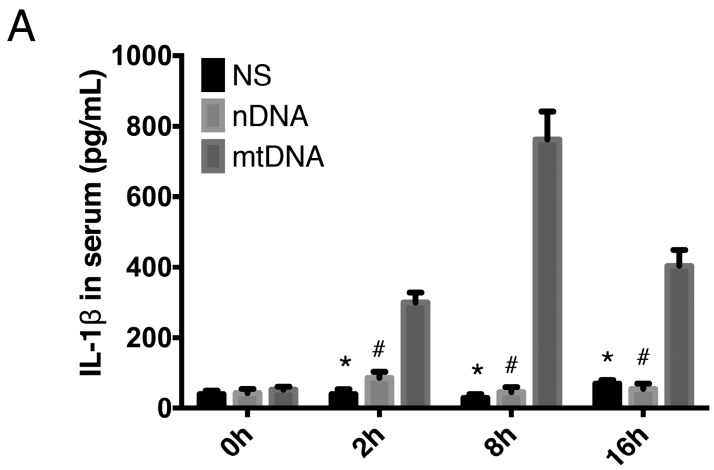

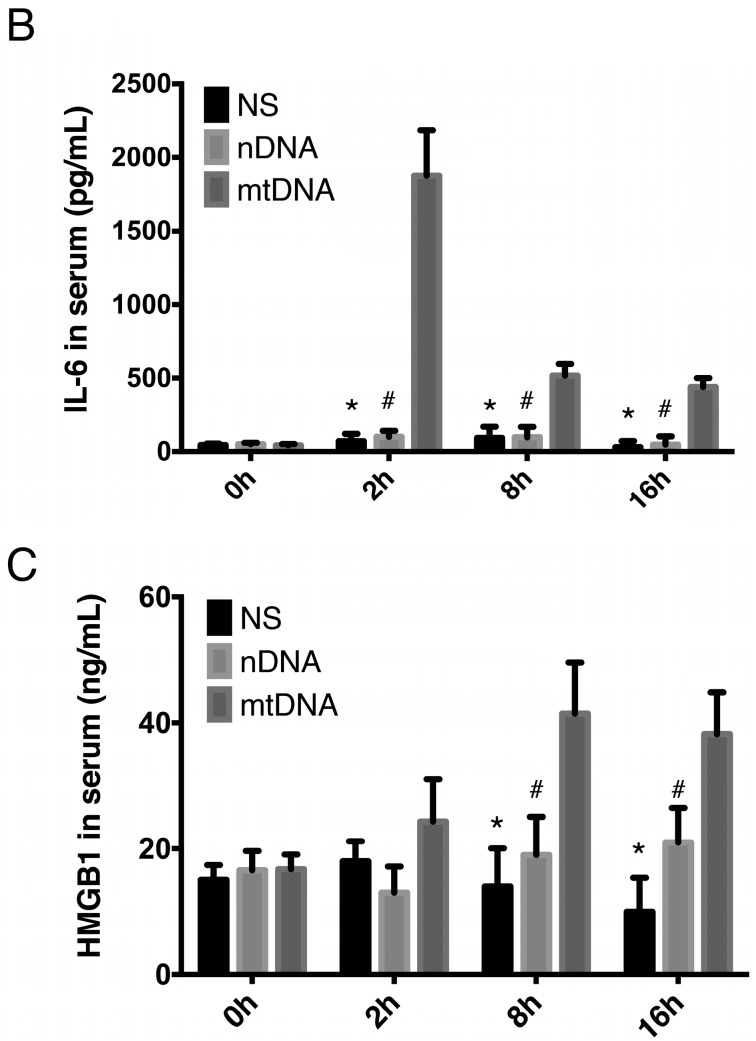
Intra-peritoneal administration of mtDNA leads to systemic inflammation. WT mice were treated with PBS, nuclear DNA (nDNA), or mtDNA via intra-peritoneal injection for 2, 8, and 24 h. Systemic inflammation and circulating levels of IL-1β (**A**); IL-6 (**B**); and HMGB1 (**C**) were measured by ELISA. * *p* < 0.05 versus mtDNA group; # *p* < 0.05 versus mtDNA group. Eight mice were used in each set and data are mean ± SEM of three separate experiments.

To provide further evidence of mtDNA-induced systemic inflammation, WT mice were treated with NS, nDNA, or mtDNA via intra-peritoneal injection 2, 8, and 16 h. Circulating levels of IL-1β, IL-6, and high-mobility group protein B1 (HMGB1) were then measured by ELISA. Proinflammatory cytokines (IL-1β, IL-6, and HMGB1) were significantly higher in the mtDNA administration group compared with the levels in the nDNA and control groups (*p* < 0.05). The concentrations of IL-1β and HMGB1 peaked 8 h, and the concentration of IL-6 peaked 2 h, after mtDNA administration and then gradually decreased, however the levels were still significantly higher compared to the nDNA and control groups (Figure 3A–C). Administration of nDNA did not cause systemic inflammation (Figure 3A–C). These data indicated that mtDNA triggers a significant increase in IL-1β, IL-6, and HMGB1 production.

### 2.4. Intra-Peritoneal Administration of mtDNA Induces Acute Lung Injury and Systemic Inflammation in a TLR4-Independent Manner

**Figure 4:**
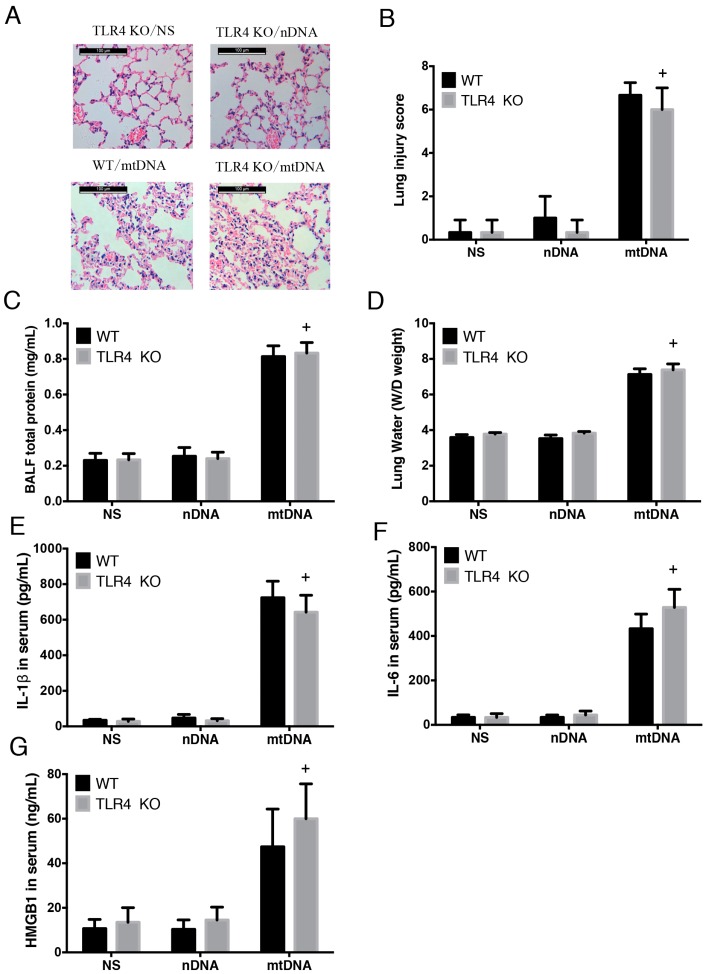
Intra-peritoneal administration of mtDNA induces acute lung injury and systemic inflammation in a TLR4-independent manner. WT and TLR4 KO mice were given NS, nDNA, and mtDNA administered intra-peritoneally (*n* = 16/group, 8 for BALF only). Eight hours later, acute lung injury was measure by HE staining (scale bar is 100 μm) (**A**) for lung injury score (**B**); BALF total protein concentration (**C**); and lung W/D ratio (**D**) were also analyzed. Systemic inflammation and circulating levels of IL-1β (**E**); IL-6 (**F**); and HMGB1 (**G**) were measured by ELISA. + *p* > 0.05 versus WT/mtDNA group. Eight mice were used in each set and data are mean ± SEM of three separate experiments.

As demonstrated above, LPS-induced mtDNA release occurs in a TLR4-dependent manner. Therefore, we further examined whether mtDNA leads to ALI and systemic inflammation via TLR4. WT and matched TLR4 KO mice were randomly assigned to sham and mtDNA groups. There were no significant differences in acute lung injury, as demonstrated by HE staining, lung injury score; BALF total protein concentration or lung W/D ratio between the two groups 8 h following mtDNA administration (Figure 4A–D). In addition, there were no significant differences in circulating levels of IL-1β, IL-6 or HMGB1 between the two groups (Figure 4E–G). This suggests that knock out of TLR4 provides no protection against mtDNA-induced ALI and systemic inflammation (*p* > 0.05) and indicates that mtDNA-induced ALI and systemic inflammation occurs in a TLR4-independent manner.

### 2.5. Intra-Peritoneal Administration of mtDNA Induces Acute Lung Injury and Systemic Inflammation in a TLR9-Dependent Manner

**Figure 5:**
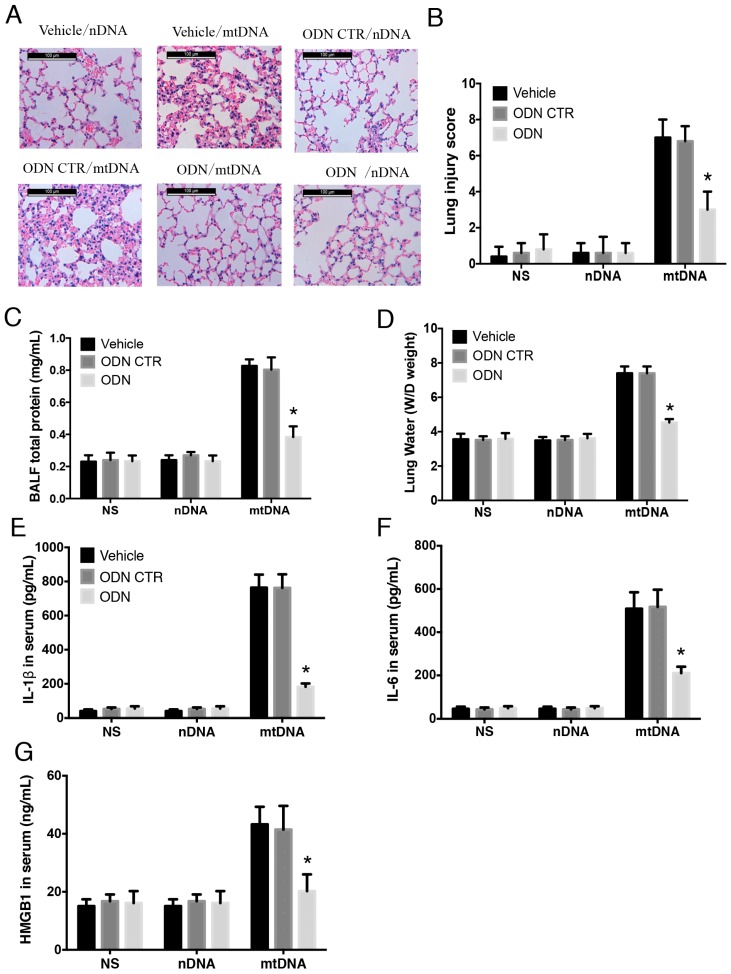
Intra-peritoneal administration of mtDNA induces acute lung injury and systemic inflammation in a TLR9-dependent manner. WT mice were randomly assigned to a group pretreated with the TLR9 specific inhibitor ODN2088 or a control group. Mice in the ODN2088 were pretreated 1 h before mtDNA administration (*n* = 16/group, 8 for BALF only). Acute lung injury was measured by HE staining (scale bar is 100 μm) (**A**) for lung injury score (**B**); BALF total protein concentration (**C**); and lung W/D ratio (**D**) were also analyzed. Systemic inflammation and circulating levels of IL-1β (**E**); IL-6 (**F**); and HMGB1 (**G**) were measured by ELISA. * *p* < 0.05 versus control group. Eight mice were used in each set and data are mean ± SEM of three separate experiments.

TLR9 is well-known toll-like pattern recognition receptor for mtDNA [32,33], thus the TLR9-specific inhibitor, ODN2088 was used to further examine whether TLR9 may be a key upstream molecule, which mediates the inflammatory responses induced by mtDNA. MtDNA was administered intraperitoneally to C57BL/6 mice, which had been pre-treated with ODN2088 or vehicle (control ODN or NS). Pre-treatment with ODN2088 significantly attenuated mtDNA-induced ALI compared to control groups, as demonstrated by improved histological changes (Figure 5A), decreased lung injury scores (Figure 5B), decreased BALF total protein concentrations (Figure 5C), and decreased lung W/D ratios (Figure 5D) (*p* < 0.05).

Circulating levels of IL-1β, IL-6, and HMGB1 were assessed to further evaluate systemic inflammation. Pretreatment with the TLR9 inhibitor resulted in a significant reduction in the concentrations of IL-6, HMGB1, and IL-1β compared with the levels found in the mtDNA-exposure group (*p* < 0.05) (Figure 5E–G). Taken together, these data suggest that TLR9 mediates the systemic inflammatory responses after mtDNA intra-peritoneal administration. MtDNA induced acute lung injury and systemic inflammation in TLR9-dependent manner.

### 2.6. Intra-Peritoneal Administration of mtDNA Leads to p38 MAPK Activation via TLR9 

**Figure 6:**
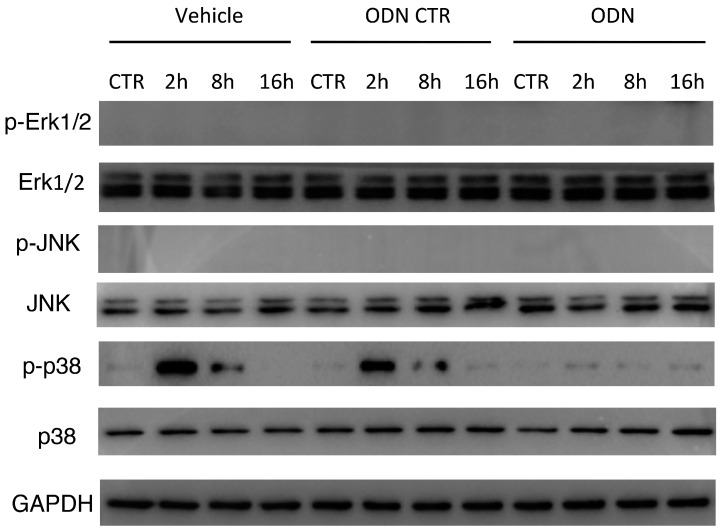
Intra-peritoneal administration of mtDNA leads to p38 MAPK activation via TLR9. WT mice were randomly assigned a group pretreated with TLR9 specific inhibitor ODN2088 or a control group. Mice in the ODN2088 group were pretreated 1h before mtDNA administration (*n* = 8/group). Total and phosphorylation levels of ERK1/2, JNK, and p38 expression in lung tissue were measured by Western blot. Three separate independent experiments got the similar results.

Considering that p38 MAPK is known as an early downstream molecule of TLR9 [9,29], Western blot was used to explore the expression of total and phosphorylated p38, JNK and Erk1/2 MAPK in lung tissues following mtDNA administration. Intra-peritoneal injection of mtDNA leads to a significant up-regulation of phosphorylated p38 MAPK at 2 h; the level returned to baseline 16 h after mtDNA administration (Figure 6). Specific TLR9 inhibitor ODN2088 pretreatment significantly suppressed mtDNA-induced p38 MAPK phosphorylation in the lung tissues compared with the control group. However, the expression of total p38 MAPK, as well as phosphorylated and total Erk1/2 MAPK and JNK, was not significantly changed following mtDNA exposure and TLR9 inhibitor pretreatment (Figure 6). These results indicate that mtDNA may induce p38 MAPK phosphorylation through TLR9 in vivo, which may be involved in mtDNA induced lung inflammatory responses.

## 3. Discussion

Despite major improvements in antibiotic therapy and critical care techniques [34], sepsis still contributes to significant morbidity and mortality in intensive care units (ICUs) [1]. Mitochondrial damage is common in septic patients and the severity of mitochondrial injury is positively correlated with the level of inflammatory factors, disease severity and prognosis [35]. The role of mitochondrial dysfunction has been involved in sepsis-induced MOF through the following mechanisms [5,6]: (1) the damage to mitochondria induces intrinsic apoptosis, which leads to immunosuppression [36]; (2) the damage to mitochondria leads to cell energy metabolism disorder [37]; and (3) the damage to mitochondria is involved in the occurrence of the systemic inflammatory response [9,38].

The latest research suggests that, in addition to ROS, damaged mitochondria release another endogenous alarmin, mtDNA, which provokes the innate immune system through a variety of signal transduction pathways, including extracellular mtDNA, synergism with ROS, activation of macrophage NALP3 inflammasomes, caspase-1 activation, and regulation of IL-1 and IL-18 processing and maturation [24,36]. Release of mtDNA into the circulation due to injury activates the neutrophil p38 MAPK signaling pathway via TLR9 and contributes to the development of post-traumatic SIRS [9]. In addition, lung histological scores were found to be significantly higher in an mtDNA intra-tracheal administration group compared with nDNA groups [30]. The current study demonstrates that intra-peritoneal administration of mtDNA may also lead to remote end organ damage and systemic inflammatory responses. Mitochondria evolved via endosymbiosis and were derived from bacteria; therefore, mitochondria possess formyl peptides and circular DNA with un-methylated repeats, like bacterial DNA [9]. The fact that mtDNA has been found to induce remote organ injury and inflammation may potentially serve as an endogenous harmful molecule, just like “Trojan horse” by analogy [39].

Yamanouchi et al. [40] found that mtDNA levels are elevated during traumatic injury and severe sepsis. After the administration of anthrax, bacterial DNA increased temporarily while mtDNA levels remained elevated until death, indicating that the damage continues after the bacteria were obliterated [21]. Our research showed that marked elevations in circulatory mtDNA were found in animal models as early as 2 h post-LPS administration and the mtDNA level peaked at 8 h following LPS administration. Data in this study indicate that mtDNA release occurs at early phase and last for hours, making it a potential therapeutic target. In addition, compared with their WT counterparts, plasma mtDNA copies in TLR4 KO mice were significantly decreased, indicating that LPS-induced circulating mtDNA occurs in both a time-dependent and TLR4-dependent manner. To the best of our knowledge, the present study provides the first report of the dynamic and mechanism of LPS-induced circulatory mtDNA release in a murine endotoxemia model. However, based on our data, TLR4 was not found to be a downstream effector of mtDNA.

Similar to bacterial DNA, mtDNA is circular, contains a higher frequency of un-methylated CpG dinucleotides and the oxidative status can be recognized by the innate immune system [41]. TLR9, localized in the endolysosome, senses DNA with un-methylated CpG motifs derived from bacteria and viruses. The ability of TLR9 to discriminate between foreign and self-DNA is due to the higher frequency and presence of un-methylated CpG dinocluoteides in bacteria and viruses compared with mammalian DNA [42]. Recently, increasing evidences have shown that mtDNA provokes the immune response directly via the activation of TLR9 as its ligand [9,43]. In this study, TLR9 inhibitor pretreatment resulted in an abatement of mtDNA-induced local lung injury and systemic inflammation. Thus, mtDNA induced ALI and inflammatory responses through TLR9.

It has been demonstrated that mtDNA plays an essential role in innate immune modulation [44,45]. Mice treated with mtDNA showed significant acute lung injury, neutrophils activation, and increased release of matrix metalloproteinase-8 (MMP-8) [43]. Furthermore, TLR9 interacted with mtDNA can activate nuclear factor kappa B (NF-κB) signaling. Many pro-inflammatory cytokines levels, such as tumor necrosis factor-α (TNF-α), interleukin (IL)-6, and IL-1β were also elevated significantly [9,29,46]. Exposing cultured macrophages to mtDNA up-regulates several proinflammatory cytokines via the TLR9–p38 MAPK signal pathway [29]. mtDNA also plays a role in increased endothelial cell permeability via increased calcium flux and MAPK phosphorylation [28]. Oxidized mtDNA released from disrupted mitochondria directly binds and activates NLRP3 inflammasomes [47]. In lung tissue, mtDNA increases NF-κB, IκB-α and TLR9 mRNA levels and also increases phosphorylated NF-κB p65 and TLR9 protein levels in macrophage cultures [48]. Data from the current study indicate that LPS-induced mtDNA release may lead to p38 MAPK activation and probably subsequent lung inflammatory responses. However, the function and mechanism of MAPK activation in mtDNA-induced end organ damage and systemic inflammation still need further study.

## 4. Materials and Methods

### 4.1. Main Reagents

LPS (*Escherichia coli* 0111:B4) was obtained from Sigma-Aldrich (St. Louis, MO, USA). TLR9 antagonist CpG (ODN2088) and control CpG were obtained from InvivoGen (San Diego, CA, USA) and suspended in vehicle (0.9% sodium chloride, NS), aliquoted and conserved at −20 °C until use. A mitochondrial isolation kit was purchased from BioVision (Milpitas, CA, USA). Rabbit phospho-p38 MAPK (Thr180/Tyr182) antibody duet, phospho-p44/42 MAPK (Erk1/2) (Thr202/Tyr204) antibody duet, phospho-SAPK/JNK antibody duet, and GAPDH antibodies were all obtained from Cell Signaling (Boston, MA, USA).

### 4.2. Animals

TLR4 KO mice were purchased from Jackson Laboratory (Bar Harbor, ME, USA). The control mice (C57BL/6J) were provided by the Laboratory Animal Center of Central South University. All mice used in this study were male, 8–10 weeks of age and weighed between 25 g and 30 g. Animals were maintained in a 12-h light/dark cycle. The Animal Care and Use Committee of the Central South University approved all animal protocols (NO.201403122). All experiments were conducted in accordance with the National Institutes of Health Guidelines for the Care and Use of Laboratory Animals.

### 4.3. In Vivo Experimental Design

For examination of LPS-induced mtDNA release, mice were randomly assigned to four groups: WT + PBS (*n* = 8), WT + LPS (*n* = 8), TLR4 KO + PBS (*n* = 8), and TLR4 KO + LPS (*n* = 8). LPS groups were given a lethal dose of LPS (20 mg/kg) administered intraperitoneally (i.p.) at the indicated time point. PBS groups received injections of sterilized PBS, administered i.p. as above. Following PBS or LPS administration, mice were euthanized and samples of blood and lung tissue were collected for further study.

C57BL/6J mice were used for the mtDNA intra-peritoneal administration experiment. nDNA (5 mg/kg), mtDNA (5 mg/kg) or 0.9% sodium chloride vehicle (normal saline, NS) was administered in a volume of 200 μL via intra-peritoneal (i.p.) injection. The DNA concentration selected was sufficient to induce SIRS, according to previous publications [9,29]. Mice were treated with ODN2088 (100 μg per mouse) or control ODN and NS via intra-peritoneal injection 1 h prior to mtDNA administration for the TLR9 specific inhibition experiment. Two, 8, and 16 h after the treatment of mtDNA, nDNA, or NS, the animals were euthanized by cervical dislocation and blood was collected and centrifuged at 2000× *g* for 5 min. The plasma was stored at −80 °C for subsequent cytokine assays. Acute lung injury was measure by HE staining for lung injury score; BALF total protein concentration and lung W/D ratio were also analyzed. Circulating levels of IL-1β, IL-6, and HMGB1 were measured by ELISA to assess systemic inflammation.

Following euthanasia, the lungs (*n* = 8 per group) were excised from the mice by opening the chest via median sternotomy. The wet weight (W) of the left upper lung lobe was measured using an electronic scale and then desiccated in an oven at 65 °C for 48 h for determination of the dry weight (D). The water content was obtained by calculating the W/D weight ratio. The left inferior lobe was removed and fixed in 4% paraformaldehyde (PFA) for 24 h. The remaining parts of left lungs were snap frozen and stored at −80 °C for subsequent protein extraction. Following euthanasia, lungs (*n* = 8 per group) were lavaged with 1 mL sterile saline each time using an intra-tracheal catheter, and a total of 2 mL broncho-alveolar lavage was instilled and withdrawn from each mouse [49,50]. The bronchoalveolar lavage fluid (BALF) was centrifuged at 500× *g* for 10 min at 4 °C and protein concentrations were determined by a bicinchoninic acid (BCA) protein assay kit (Beyotime, Shanghai, China).

### 4.4. Preparation of mtDNA and nDNA

Mitochondria were isolated from the liver of wildtype C57BL/6 mice using a mitochondrial isolation kit (BioVision) following the manufacturer’s instructions. The nuclear fractions of hepatocytes were reserved for the subsequent preparation of nDNA.

MtDNA and nDNA were purified from mitochondrial pellets and nuclear fractions respectively using DNeasy blood and tissue kits (Qiagen, Hilden, Germany). Both mtDNA and nDNA were dissolved in sterilized 0.9% sodium chloride (NS). The DNA concentrations and purity were analyzed using a NanoDrop 2000 (Thermo Fisher Scientific, Waltham, MA, USA). The quality of all DNA samples was checked on a NanoDrop2000 (Thermo Fisher Scientific, MA, USA). Furthermore, we confirmed that no detectable endotoxin levels in the samples (Associates of Cape Cod Inc., East Falmouth, MA, USA). To further exclude any significant nDNA contamination and to ensure the purity of the mtDNA, Q-PCR was conducted. The nDNA content was less than 0.1% in the isolated mtDNA samples.

### 4.5. Hematoxylin and Eosin Staining

A portion of lung from the left lobe of each mouse was washed twice with PBS and fixed in 4% paraformaldehyde at 4 °C for 24 h. Then, the lungs were embedded in paraffin. Sections (5 µm thick) were affixed to slides and stained. Histopathological changes in the slices were observed with a microscope and pathological changes were evaluated in a double blind fashion. The severity of acute lung injury was evaluated using a semi-quantitative histological index, as described previously [51].

### 4.6. Quantification of Inflammatory Cytokines

IL-1β and IL-6 plasma 1evels were determined using a commercially available mouse ELISA kit (R and D Systems, Minneapolis, MN, USA), according to the manufacturer’s instructions. HMGB1 levels in all samples were determined using an HMGB1 Detection Kit (Chondrex Inc., Redmond, WA, USA), according to the manufacturer’s instructions.

### 4.7. Circulating DNA Purification And Quantitative Real-Time PCR

Circulating DNA was isolated from 250 μL plasma using an ENZA Circulating DNA Kit (Omega Bio-Tek, Norcross, GA, USA) according to the manufacturer’s protocol. MtDNA (cytochrome c oxidase 1, mtCOI) copy number was measured by quantitative PCR using SYBR Green PCR Mix (GeneCopoeia, Shanghai, China) and normalized to nuclear DNA (18s RNA) levels. Two independent reactions were performed [52]. The following primers (Sangon Corp., Shanghai, China) were used:

18S forward, 5′-TAGAGGGACAAGTGGCGTTC-3′;
18S reverse, 5′-CGCTGAGCCAGTCAGTGT-3′;
mtCOI forward, 5′-GCCCCAGATATAGCATTCCC-3′;
mtCOI reverse, 5′-GTTCATCCTGTTCCT GCTCC-3′.

Reactions were carried out in a 20 μL final volume, containing 0.2 μM of each forward and reverse primer, 20 ng DNA sample, and 10 μL SYBR Green PCR Mix. Amplification was performed using an Applied Biosystems 7300 Real-Time PCR machine (Life Technologies, MA, USA) under a thermal profile of 95 °C for 10 min followed by 40 cycles at 95 °C for 15 s, 60 °C for 20 s and 72 °C for 20 s. The threshold cycle (*C*_t_) was obtained from duplicate samples and averaged. Target gene levels were determined using the ΔΔ*C*_t_ method and expressed as relative to the control group, using the formula: ΔΔ*C*_t_ = ((*C*_t_ gene of interest − *C*_t_ internal control)·sample) – ((*C*_t_ gene of interest − *C*_t_ internal control)·control).

### 4.8. Tissue Protein Extraction and Western Blotting

The collected lungs were homogenized, resuspended with RIPA lysis buffer containing proteinase inhibitors. After sonication, the lysates were centrifuged at 12,000× *g* for 10 min at 4 °C. Protein concentrations were quantitated with a bicinchoninic acid (BCA) protein assay kit (Beyotime, Shanghai, China) and 25 µg of protein per sample was mixed with sample loading buffer and boiled for 5 min at 95 °C. Proteins were separated in 12% sodium dodecyl sulfate-polyacrylamide gel (SDS-PAGE) and transferred onto polyvinylidene fluoride (PVDF) membranes (Millipore, Billerica, MA, USA). After blocking with 3% bovine serum albumin (BSA) for 1 h at room temperature, the membranes were then incubated with primary antibodies against p38 (1:1000), p-p38 (1:1000), Erk1/2 (1:2000), p-Erk1/2 (1:1000), JNK (1:2000), p-JNK (1:2000), and GAPDH (1:2000) overnight at 4 °C. Washed three times (10 min each) with TBS-T, membranes were then incubated with secondary antibody conjugated with horseradish peroxidase (HRP) for 1 h at room temperature. The blots were imaged using a ChemiDoc MP imaging system (Bio-Rad Laboratories, Berkeley, CA, USA) and quantified with ImageJ software version 1.49 (National Institutes of Health, Bethesda, MD, USA).

### 4.9. Statistical Analyses

All data are expressed as mean ± standard error of mean (SEM). Significant differences in the same group were analyzed using repeated measures analysis of variance (ANOVA) followed by the least significant difference (LSD) test. Significant differences between groups were determined by one-way ANOVA; *p* < 0.05 was considered statistically significant. All calculations and statistical analyses were performed using Statistical Product and Service Solutions （SPSS）software (version 17.0) (International Business Machines Corporation, Armonk, NY, USA) for Windows.

## 5. Conclusions

In conclusion, the present study demonstrates that mtDNA, as a novel DAMP, is released into the extracellular milieu, in response to LPS stimulation, in a TLR4-dependent manner. Intra-peritoneal administration of mtDNA induces a systemic inflammatory response, partly through TLR9 signaling. MtDNA, as a novel mitochondrial DAMP, may independently trigger lung inflammation and play an important etiological role in sepsis. Therefore, suppressive oligodeoxynucleotides and anti-TLR9 treatment, which minimize or counteract the impact of extracellular mtDNA, may be promising therapies for sepsis treatment.
